# Glyphosate Use in Crop Systems: Risks to Health and Sustainable Alternatives

**DOI:** 10.3390/toxics13110971

**Published:** 2025-11-12

**Authors:** Pamela G. Aoun, Walid Khairallah, Abderahman Rejeb, Amira Haddarah

**Affiliations:** 1 Doctoral School of Sciences and Technology, Lebanese University, Rafic Hariri Campus, Hadath 1533, Lebanon; pamela.g.aoun@gmail.com; 2 Endocrinology Bellevue Medical Center, Medical Director Green Clinics, Mansourieh 22411, Lebanon; drwk@greenclinics.org; 3 Kautz Faculty of Business and Economics, Széchenyi István University, 9026 Győr, Hungary

**Keywords:** glyphosate, herbicide impacts, environmental sustainability, human health risks, sustainable development goals (SDGs), weed management alternatives

## Abstract

Glyphosate, a widely used non-selective herbicide, has been a subject of intense scientific debate due to its environmental persistence and potential health risks. This review examines glyphosate’s mechanisms of action, its effects on crop production, and its broader environmental impact, including soil degradation, water contamination, and biodiversity loss. Furthermore, it examines the expanding body of research linking glyphosate exposure to various human health concerns, including metabolic, neurological, reproductive, and oncological disorders. The review also assesses glyphosate’s role in hindering the achievement of the Sustainable Development Goals (SDGs), particularly those related to food security, health, access to clean water, and the protection of marine ecosystems. Finally, potential alternatives to glyphosate-based weed control, including organic and non-chemical methods, are discussed to promote sustainable agricultural practices that balance productivity with ecological and public health considerations. The evidence reviewed highlights glyphosate’s pervasive presence across ecosystems and its potential to disrupt both environmental and human health. The findings underscore the urgent need to regulate glyphosate use, prioritize soil and water protection, and accelerate the transition toward sustainable, low-toxicity weed management strategies that align with global sustainability objectives.

## 1. The Introduction

Glyphosate, an organic chemical compound with the formula C_3_H_8_NO_5_P, is marketed under a long list of names, including RoundUp, RoundUp Pro, RoundUp Ultra, Aqua Neat, AquaMaster, Accord, Rodeo, Polado, Touchdown, EZ-Ject, Glyfos, Expedite, Laredo, Wrangler, and Buccaneer Plus, among others. While these products contain a variety of chemical formulations, Glyphosate remains the primary active ingredient, accounting for 36 to 48% of the product content [1].

Glyphosate, known as *N*-(phosphonomethyl)-glycine, is a powerful non-selective systemic herbicide derived from phosphonic acid and glycine. Initially discovered in 1950, it gained widespread commercialization under the brand name of RoundUp by Monsanto [2]. Its usage has skyrocketed globally, projected to escalate from an annual range of 600 to 750 thousand tonnes to approximately 920 thousand tonnes by 2025 [3]. In 2014, glyphosate use in the United States had risen to 125 million kilograms, an increase of 15-fold since the 1970s. In 2021, nearly 150,000 tons of glyphosate were sprayed onto American crops, equivalent to one pound of glyphosate per person in a year [1]. The dosage reported refers to the active ingredient of glyphosate rather than the formulated product.

In 1961, glyphosate was patented by the Stauffer Chemical Company and was used as a chelating agent. In 1968, it was taken up by Monsanto and was used as a herbicide, marking a totally different application. In the early 2000s, it was patented a 3rd time by Monsanto, this time as an oral antibiotic [1].

Despite its relatively low harm to animals, glyphosate has ignited substantial debate and concern due to its extensive usage and associated properties. It possesses high water solubility, sorption properties, and low hydrophobicity and volatility, making it a persistent environmental contaminant that necessitates complex analytical methods for detection [4,5].

Research interest in glyphosate has steadily increased since 1975, reaching its peak in the 1990s with the emergence of genetically modified crops and has experienced renewed attention since 2015, following its classification as “probably carcinogenic” to humans (Group 2A) by the International Agency for Research on Cancer [6,7,8,9].

## 2. Glyphosate Mechanism

Generally applied to the foliage of weeds, glyphosate can infiltrate plants through four potential pathways: the leaves or other green tissues, the roots, the trunk, or shoots emerging from the root &/or trunk [10]. Once inside the plants, it swiftly moves to regions of active growth.

Both adsorption and desorption in soil, along with other physical, chemical, and biological factors, affect the biodegradation rate of glyphosate. Glyphosate degradation can occur under both aerobic and anaerobic conditions, although it tends to be slower in anaerobic environments compared to aerobic ones [11]. Additionally, the temperature of the soil and the amount of phosphate present can affect the degradation rate of glyphosate [12,13].

Glyphosate, despite its high solubility in water, exhibits limited movement within the soil profile due to its strong adsorption to soil particles [14]. Factors influencing glyphosate adsorption include clay content, organic matter, and the presence of iron and aluminum oxides in the soil. Soil processes, such as adsorption and desorption, play a crucial role in determining glyphosate degradation rates, with strong adsorption potentially hindering microbial access to the compound. For instance, Sorensen et al. [15] observed limited glyphosate bioavailability in deeper layers of sandy soil due to high adsorption and low desorption rates, leading to minimal mineralization. However, ref. [16] found that while glyphosate adsorbed to soil particles, it remained microbially degradable, although with reduced microbial activity in its presence. While several studies have examined glyphosate adsorption characteristics, only a few have investigated its impact on glyphosate bioavailability in soil [17].

**Figure 1:**
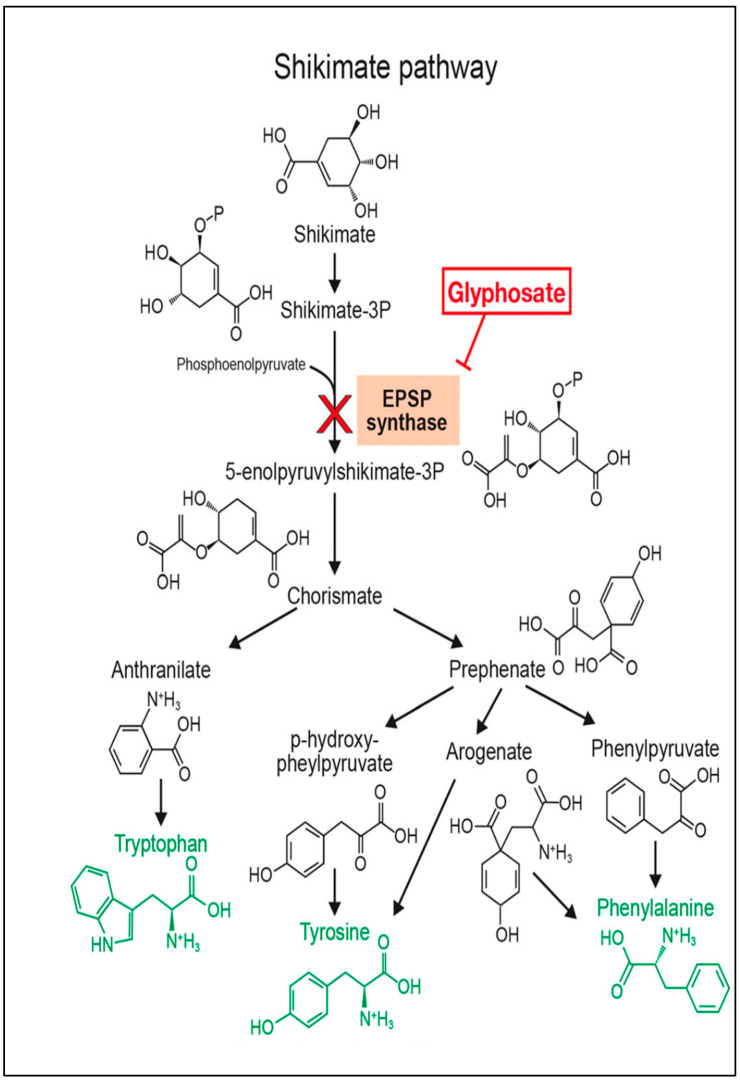
Shikimate pathway hindered by Glyphosate which acts as an inhibitor of 5-enolpyruvylshikimate-3-phosphate synthase (EPSPS) and consequently prevents the synthesis of aromatic amino acids, such as phenylalanine, tyrosine, and tryptophan [19].

Glyphosate functions as a competitive inhibitor of the shikimate pathway (check Figure 1) in microorganisms, parasites, and plants, thereby hindering the activity of the enzyme 5-enolpyruvylshikimate-3-phosphate synthase (EPSPS), which catalyzes the sixth step in the shikimate pathway [18,19]. EPSPS normally facilitates the reaction between shikimate-3-phosphate (S3P) and phosphoenolpyruvate (PEP) to form 5-enolpyruvylshikimate-3-phosphate (EPSP). Glyphosate acts as a structural analog of PEP and binds tightly to the EPSPS active site, forming a stable enzyme–substrate complex that blocks access of PEP. This inhibition prevents the synthesis of aromatic amino acids, such as phenylalanine, tyrosine, and tryptophan, which are produced through the shikimate pathway [20]. Consequently, the metabolic flow through the shikimate pathway is interrupted, leading to depletion of these amino acids, disruption of protein synthesis, and inhibition of secondary metabolites required for plant growth, defense, and pigment production. This pathway is critical for the production and synthesis of several pigments, including anthocyanins and flavonoids [21].

Despite extensive research, over a thousand molecules that share similarities with glyphosate in terms of shape and biophysical properties have been examined. However, none have matched glyphosate’s ability to suppress EPSP synthase in the shikimate pathway. This underscores the unique and unparalleled efficacy of glyphosate in its mode of action [22].

## 3. Potential Effects of Glyphosate in Crop Production

The three major concerns regarding the extensive use of glyphosate are its effects on crop health, its interaction with crop nutrition, and its persistence in the environment [23].

### 3.1. Effects on Crop Health

Typically, plants treated with glyphosate perish within 1 to 3 weeks, as its distribution within the plant is uniform, leading to the death of all plant parts [24]. The adverse impacts of glyphosate on non-target plants are particularly worrisome for farmers. Glyphosate, when used to manage weeds, can inadvertently affect non-target areas through multiple pathways.

#### 3.1.1. Spray Drift

The main pathway is unintentional spray applications, known as “spray drift,” which can transport the herbicide directly to unintended crops. Studies have shown that the off-target movement or drift of glyphosate during application can amount to as much as 10% of the applied dosage in crops [25,26].

#### 3.1.2. Uptake from Soil

Another possible pathway for the buildup and persistence of glyphosate in soil involves its release from plant remnants of treated weeds. Given glyphosate’s inherent stability and slow breakdown in numerous plant species, significant quantities can be efficiently transported to areas of active growth and accumulate, especially in developing tissues [27]. Once weeds perish, glyphosate enters the soil through the decomposition of the plant. Further detailed assessments have shown that glyphosate moves within plants, gathers in roots, and ultimately leaches into the rhizosphere [23].

Following the initial application, glyphosate can also be reabsorbed by both target and non-target plants through their roots, originating from the soil. Several studies have explored the impact of glyphosate exposure in the root zone on crops such as cotton, maize, and rapeseed, suggesting that crop roots can absorb glyphosate. However, most of these conclusions were drawn from observations in hydroponic nutrient solutions. Therefore, further research is needed to enhance our understanding of glyphosate uptake from soils and its subsequent effects on crop performance [23].

#### 3.1.3. Inactivation of EPSPS

Glyphosate disrupts the synthesis of multiple phenolic compounds derived from the shikimate pathway, which are responsible for improving plant immunity. By inactivating EPSPS, glyphosate increases the plant’s susceptibility to diseases [28,29,30]. The overuse of glyphosate has been linked to disease onset in numerous crops. For example, glyphosate applications have been identified as a primary contributor to the emergence of diseases like Fusarium head blight in agronomic crops. Reports have documented elevated pathogen colonization in the roots of wheat and barley following glyphosate burndown applications before planting [31].

### 3.2. Interaction with Crop Nutrition

Glyphosate interacts with soil either through direct contact from foliar sprays reaching the soil surface or from decomposing weed tissue. Once in the soil, glyphosate binds to soil colloids, leading to its persistence. Unlike many other herbicides, glyphosate adsorption onto soil is primarily influenced by soil minerals rather than organic matter [32]. Recent assessments have emphasized glyphosate’s role in contributing to nutrient deficiencies in crops, particularly in agricultural systems that heavily rely on it for weed control [23,33]. Glyphosate acts as a chelator of divalent metal cations, including zinc, copper, manganese, cobalt, and iron, which can potentially hinder nutrient uptake and translocation in crops. As a result, plants exposed to glyphosate absorb lower quantities of these essential minerals into their tissues. Consequently, foods derived from such plants may lack adequate mineral content. While humans require only minimal amounts of these minerals in their diet, deficiencies can lead to significant adverse health effects [32].

Studies suggest that glyphosate residues or drift may reduce the uptake and translocation of micronutrients, such as manganese and iron, in non-target plants. This reduction is attributed to the formation of poorly soluble chelated complexes between glyphosate and micronutrients, such as manganese, iron, zinc, and boron, in plants, whether through spray drift or root uptake. Glyphosate may influence nutrient absorption in crops, particularly of manganese and phosphorus, potentially affecting growth. Several studies support this trend, though specific effects vary with soil type and application rates [34,35,36]. Such interactions with plant nutrition could potentially affect crop health, particularly in tree crops like citrus, where micronutrients play a critical role in disease resistance mechanisms [37]. Glyphosate exposure has been associated with reduced disease resistance in crops, including increased susceptibility to Fusarium spp. and other soil-borne pathogens [32].

Glyphosate’s binding mechanism to soil solids and adsorption sites is similar to that of phosphate compounds, affecting the mobility of phosphorus in soil. The competition between glyphosate and phosphorus for adsorption sites in soil significantly affects the mobility and availability of phosphorus as a crop nutrient [38]. However, there is limited literature demonstrating the significant effect of this competition on phosphorus nutrition in crops, necessitating further investigation [23].

Moreover, as a metal chelating agent, glyphosate reduces the bioavailability of several nutrients in plants, emphasizing the necessity for integrating management strategies to mitigate nutrient loss in glyphosate-treated crops [39].

### 3.3. Persistence in the Environment

When applied as a foliar spray for weed control, glyphosate can migrate into various soil compartments and unintended areas. This can happen through processes such as wash-off from foliage, unintentional spray drift, the decomposition of plant residues treated with glyphosate, and the exudation from roots. Additionally, glyphosate may be released through exudates from the undamaged roots of glyphosate-tolerant crops [40].

A study conducted at a Danish field site investigating glyphosate movement and leaching found that, despite its strong tendency to bind to soil, glyphosate was able to penetrate deep into the soil and leach out with drainage water. Additionally, numerous water monitoring reports have highlighted the presence of glyphosate in groundwater. For instance, glyphosate was detected in 36% of 154 water samples collected from Midwestern U.S. states, where it is extensively used on corn fields. However, the concentrations found in these samples were well below the maximum contaminant level for glyphosate [23]. Beyond groundwater, glyphosate has also been identified in surface water sources, likely due to runoff [41]. Glyphosate residues were found in USA and European croplands, as well as in drains, lakes, groundwater, ponds, and ditches [42]. It was also found in air, water, soil, and food products [43]. In addition to its chemical persistence, glyphosate can affect soil-dwelling organisms that play crucial roles in nutrient cycling and soil structure. Studies have reported alterations in earthworm activity and microbial diversity following glyphosate exposure, potentially influencing soil fertility and ecosystem balance [44].

When glyphosate is dispersed in the environment, it undergoes two primary processes. The majority of it is broken down by soil microbial communities, resulting in the formation of α-amino-3-hydroxy-5-methyl-4-isoxazolepropionic acid (AMPA), its primary metabolite. Additionally, a portion is converted into sarcosine, which is then converted into glycine. Both pathways ultimately lead to the mineralization of glyphosate into carbon dioxide (CO_2_), phosphonic acid, and ammonium (NH4^+^) [45,46].

Glyphosate degrades into its metabolite AMPA, α-amino-3-hydroxy-5-methyl-4-isoxazolepropionic acid, which is transported via the phloem. It penetrates through the plant’s cuticle and is taken up by the symplast. Its movement is influenced by plant development and environmental conditions, such as soil humidity and moisture [2,4]. Its efficacy depends on its dosage and translocation within the plant. AMPA is less toxic than glyphosate, is more persistent in the environment, and has a longer half-life [47].

## 4. Potential Effects of Glyphosate on Human Health

Glyphosate has been shown to contribute to several metabolic, neurological, reproductive, oncological, and autoimmune diseases. It was once touted as remarkably safe, as its mechanism of toxicity affects the metabolic shikimate pathway found only in plants and not in humans. However, while human cells do not possess the shikimate pathway, most of our gut microbes do. These microbes are essential for providing nutrients, aiding digestion, maintaining a healthy gut barrier, and fostering the development of a robust immune system [1].

### 4.1. Glyphosate and Gut Health

Glyphosate may disrupt the enzymatic activity of trypsin and pepsin, which are responsible for protein digestion, as well as lipase, which aids in fat digestion. Consequently, undigested proteins may reach the colon, where they are metabolized by gut microbes, releasing ammonia. This disruption in protein and fat digestion could have significant implications for gut health and overall physiological function [48].

In addition, intestinal paralysis, accompanied by constipation, represents one of the severe reactions to acute glyphosate exposure. Chronic exposure to glyphosate can also lead to similar issues in the digestive tract, although the onset is typically slower, and the severity is comparatively lower [49].

Moreover, glyphosate disrupts gut pH and decreases acetate levels, likely by interfering with the synthesis of acetic acid by gut microbes. Acetic acid serves as a precursor to acetyl coenzyme A, which fuels cellular energy production via the citric acid cycle [1]. It also promotes the overgrowth of yeast in the body, potentially disturbing gut health and overall microbial balance, leading to gut dysbiosis [1].

In 2018, researchers from Italy, Denmark, and the United States conducted an experiment involving albino rats exposed to glyphosate and Roundup at doses considered safe for human consumption. Subsequently, they analyzed the rats’ microbiomes and found significant disruptions in both the mother rats and their offspring, with the pups exhibiting more severe effects than the dams. Specifically, the exposed rats displayed a decrease in beneficial bacteria, such as *Lactobacillus* and *Bifidobacteria*, and an increase in pathogenic bacteria, notably *Prevotella*, which is often linked to infections. *Lactobacillus*, crucial for early gut colonization and milk metabolism, was particularly affected, allowing pathogenic bacteria to proliferate [50].

### 4.2. Glyphosate and Liver Health

The liver, often underappreciated and misunderstood, serves as the body’s filtration system. Situated on the right side of the abdomen beneath the protective rib cage, it is a meaty, reddish organ with vital functions. The liver performs approximately 500 tasks, primarily related to metabolism, regulating vitamins and hormones, and detoxification processes. Among its many roles, the liver produces bile acids for the digestion of fats, regulates blood sugar levels through gluconeogenesis, and metabolizes toxic chemicals using cytochrome P450 enzymes. These toxins are then conjugated with small molecules, such as glutathione or sulfate, to facilitate their removal via the bloodstream and excretion in urine. Furthermore, the liver manages blood levels of vitamins and hormones, activating vitamin D, metabolizing vitamin A, and regulating thyroid hormone levels. Remarkably, the liver is the only organ capable of regeneration in the human body. Unlike other organs that form scars when damaged, the liver can replace damaged tissue with new liver cells, highlighting its exceptional healing capacity [51]. Unfortunately, the liver is among the organs most susceptible to the effects of glyphosate. Numerous studies conducted over decades have consistently shown that glyphosate is highly toxic to the liver, often manifesting early in exposure. Given its role in detoxification, the liver bears a significant burden of glyphosate exposure, as it works to clear toxins from the bloodstream [52].

Scientific research has elucidated various mechanisms through which glyphosate disrupts liver function. Glyphosate interferes with cytochrome P450 (CYP) enzymes, depletes glutathione levels, and induces oxidative stress, leading to mitochondrial dysfunction and damage to mitochondrial DNA. Additionally, glyphosate exposure contributes to the development of fatty liver disease. These findings underscore the detrimental effects of glyphosate on liver health and highlight the importance of mitigating exposure to this pervasive herbicide [52].

In a study conducted in 2020, individuals diagnosed with biopsy-confirmed nonalcoholic steatohepatitis (NASH) exhibited notably elevated levels of glyphosate residues in their urine compared to individuals without liver disease. Moreover, those with advanced-stage NASH showed higher glyphosate levels than those in the early stages of the disease. Furthermore, patients with advanced fibrosis, characterized by severe scarring in the liver, had significantly higher glyphosate levels compared to patients with less severe liver disease. These findings suggest a potential association between glyphosate exposure and the severity of liver disease, highlighting the need for further investigation into the impact of glyphosate on liver health [53].

### 4.3. Glyphosate and Brain Health

As previously highlighted, glyphosate has multiple side effects on the gut, and numerous modern diseases, including Alzheimer’s disease, amyotrophic lateral sclerosis (ALS), autism, depression, Parkinson’s disease, and rheumatoid arthritis, are now thought to originate in the gut. Gut microbes play a pivotal role in generating neurons in the hippocampus, which is crucial for brain development and neurogenesis [1].

A study conducted by researchers in Spain has revealed that glyphosate can cross the blood–brain barrier and excessively stimulate neurotransmitter receptors within various brain regions. This stimulation leads to neuronal excitotoxicity, a harmful process that results in damage and death of neurons. Upon investigating varying levels of exposure, the researchers observed a dose–response correlation between glyphosate exposure and neurotransmitter activity across different brain regions [54].

Glyphosate causes neurotoxic effects on the hippocampus by excessively stimulating NMDA (*N*-methyl-d-aspartate) receptors. When NMDA receptors are overstimulated, cells take up excessive amounts of the neurotransmitter glutamate, which acts as an excitotoxin, causing neurons to fire rapidly, ultimately resulting in cellular damage. Additionally, glyphosate can exploit glutamate transporters to enter cells, exacerbating this effect. According to a comprehensive 2019 review, glyphosate’s impact on the brain goes beyond inducing gut dysbiosis, depression, and anxiety. It likely directly damages the hippocampus through glutamate excitotoxicity. The hippocampus, along with the subventricular zone, is a key site where new neurons mature from stem cells and migrate to different brain regions. Studies on mouse neural stem cells have revealed that glyphosate significantly inhibits cell migration and differentiation, suppresses the activity of cytoprotective CYP enzymes, and triggers oxidative stress markers. This research, published in 2020, suggests that glyphosate disrupts the maturation of new neurons in the brain [55,56,57].

There are three potential mechanisms through which glyphosate formulations and glyphosate itself could induce neuroexcitotoxicity in the hippocampus:

(1) Chelation of manganese: Glyphosate may chelate manganese, rendering it unavailable as a cofactor for glutamine synthetase, an enzyme crucial for glutamate metabolism.
(2) Incorporation into glutamine synthetase: Glyphosate could incorporate into glutamine synthetase, replacing glycine at the ATP binding site, which could impair the enzyme’s function.
(3) Substitution for glycine at NMDA receptors: Glyphosate may substitute for glycine as a neurotransmitter, leading to overstimulation of NMDA receptors and excessive glutamate uptake by cells.

Furthermore, glyphosate appears to suppress the activity of acetylcholinesterase, a mechanism similar to the neurotoxic effects of organophosphate insecticides. Although the precise mechanisms are still being studied, mounting scientific evidence indicates that glyphosate is detrimental to brain health [1]. Additionally, glyphosate exposure has been linked to deficiencies in essential nutrients such as cobalamin (vitamin B12) and sulfate, which are crucial for maintaining brain health. It can also induce imbalances in short-chain fatty acids in the gut, which may affect neurological function and exacerbate inflammation [57].

The case of Autism:

It is becoming evident that brain impairment is the result of a complex interplay between genetics and the environment. Autism has been linked to a wide range of concurrent health issues, including gut dysbiosis, hormone imbalances, mineral deficiencies, heavy metal poisoning, disturbances in metabolites, and indicators of inflammation [58].

Nevison pinpointed three harmful environmental exposures that show a strong association with autism: aluminum, polybrominated diphenyl ethers (PBDEs, chemicals used as flame retardants), and glyphosate. These are considered probable contributors. Aluminum is known to be toxic to the nervous system. When introduced into the body via injection as a vaccine adjuvant, it is absorbed by macrophages, which then transport it to affected tissues, including the brain [59].

Glyphosate facilitates the transport of aluminum across the blood–brain barrier. By chelating with aluminum, glyphosate neutralizes its +3 charge, allowing the aluminum-glyphosate complex to pass through barriers more easily [60].

The most extensive recent study on pesticides and autism was conducted in California’s San Joaquin Valley, a major agricultural region. Researchers investigated pesticides and herbicides, including chlorpyrifos and glyphosate. They discovered that pregnant women living within 1.2 miles of areas where these chemicals were sprayed were 30 percent more likely to have children with severe autism. An odds ratio measures the contribution of a specific factor to a disease, with a higher ratio indicating a stronger effect. When analyzing multiple chemicals, researchers can statistically isolate the effects of each chemical relative to others. In this study, after adjusting for simultaneous exposure to other pesticides, many of the initially elevated odds ratios decreased, but glyphosate’s odds ratio actually increased. The highest odds ratio was observed among children exposed to glyphosate during their first year of life [61].

### 4.4. Glyphosate and Respiratory System’s Health

Glyphosate has been identified as a contributing factor to the rising rates of lung damage, asthma, and allergies. Additionally, concurrent exposure to low-dose glyphosate and various antibiotics can have catastrophic effects. Chronic exposure to glyphosate, which acts as an antibiotic, may increase the susceptibility of humans to respiratory infections, such as the flu and COVID-19 [1].

### 4.5. Glyphosate and Endocrine System’s Health

Glyphosate has been found to exhibit 8 out of 10 key characteristics of an endocrine disruptor. Specifically, it has been shown to disrupt thyroid hormone regulation, suppress testosterone synthesis, inhibit aromatase (an enzyme critical for development that converts testosterone to estrogen), and act as an estrogen receptor to enhance estrogenic signaling in breast cancer cells, among other effects [1].

A discovery made by Brazilian scientists in 2003 revealed that glyphosate-based formulations, such as Roundup, can disrupt the endocrine system [62]. Many toxic chemicals act as endocrine disruptors, interfering with hormone signaling by binding to receptors, signaling falsely, or disrupting hormone synthesis or transport. Surprisingly, these disruptors can be more harmful at lower doses, challenging the conventional belief that toxicity increases with dosage. Lower doses of endocrine disruptors have been associated with infertility and pose heightened risks during pregnancy, leading to complications, miscarriage, and birth defects. Additionally, the timing of exposure during pregnancy plays a crucial role in determining its effects [63,64]. Glyphosate-based herbicides have been identified as endocrine disruptors that can decrease fertility when offspring are exposed during critical moments of their development [1].

In a 2018 study conducted in Greece, human sperm exposed in vitro to a relatively low concentration of 1 mg/L of Roundup exhibited deleterious effects on sperm motility, which were associated with mitochondrial dysfunction [65]. In addition, researchers have discovered that crabs exposed to glyphosate experience decreased sperm counts and an elevated presence of abnormal sperm [66].

Leydig cells play a crucial role in testosterone production, and Roundup has been shown to inhibit hormone production in these cells by disrupting the function of the steroidogenic acute regulatory protein (StAR), which is essential for testosterone synthesis [67]. The disruption of aromatase by glyphosate is a significant concern due to its implications for maintaining a balanced sex hormone system. Aromatase is crucial for converting testosterone to estrogen, and any interference with its activity can lead to an imbalance in sex hormone levels [68]. Studies have demonstrated that glyphosate can directly suppress the activity of aromatase [68]. For example, human placental cells exposed to Roundup at concentrations much lower than those typically used in agriculture showed disrupted aromatase activity. This disruption prevents the conversion of testosterone to estrogen, leading to an accumulation of testosterone and a deficiency of estrogen. The imbalance in sex hormones caused by glyphosate’s effects on aromatase can have far-reaching consequences for reproductive health, development, and overall physiological function. Further research into the mechanisms and effects of glyphosate on aromatase activity is warranted to better understand its implications for human health [68].

Glyphosate-exposed rats suffered from various adverse health outcomes, including hormonal imbalances and reproductive issues. Female rats, in particular, showed abnormally elevated levels of testosterone [50]. Moreover, a study conducted on pregnant women in Indiana revealed an association between glyphosate levels in urine and premature birth. This finding underscores the potential adverse effects of glyphosate exposure on reproductive health and pregnancy outcomes [69].

Glyphosate can traverse the placenta, reaching the baby’s body, as evidenced by its presence in umbilical cord blood. Furthermore, it can permeate into breast milk, leading to its detection in the serum of offspring exposed to maternal sources of glyphosate [70,71].

#### 4.5.1. Glyphosate and Thyroid Health

A study involving over 35,000 individuals demonstrated an elevated risk of hypothyroidism among those highly exposed to various pesticides, including glyphosate. Thyroid hormone synthesis relies on tyrosine, a product of the shikimate pathway. Glyphosate’s disruption of the gut microbiome is believed to reduce the availability of tyrosine, which may lead to inadequate thyroid hormone production. This highlights a potential mechanism through which glyphosate exposure may contribute to thyroid dysfunction and hypothyroidism [72].

#### 4.5.2. Glyphosate and Polycystic Ovary Syndrome

Polycystic ovary syndrome (PCOS) is characterized by the accumulation of cysts on the ovaries, which are actually small, fluid-filled sacs containing immature eggs. These cysts form when the ovaries fail to release an egg during the menstrual cycle, leading to the development of multiple cysts over time. PCOS is estimated to affect around 5–10% of women of reproductive age worldwide, making it one of the most common endocrine disorders in women. However, the exact prevalence may vary depending on the population studied and the diagnostic criteria used [73]. PCOS is characterized by a range of symptoms, including irregular menstrual cycles, excess hair growth (hirsutism), acne, and skin discoloration known as acanthosis nigricans. Infertility is a common complication of PCOS [74]. Women with PCOS often exhibit increased levels of androgens (male hormones) such as androstenedione and testosterone, a condition known as hyperandrogenism. This hormonal imbalance can lead to symptoms such as irregular menstrual cycles, hirsutism (excess hair growth in a male pattern), and acne. Additionally, PCOS is frequently associated with metabolic disturbances, including obesity, insulin resistance, and an elevated risk of developing type 2 diabetes and cardiovascular disease. These metabolic features are often collectively referred to as metabolic syndrome [75]. Furthermore, studies have suggested a potential link between PCOS and an increased risk of having children with autism spectrum disorder (ASD). However, the exact mechanisms underlying this association remain unclear and require further investigation [76].

There is ongoing research into potential factors contributing to PCOS, and some scientists have proposed that disruptions in the gut microbiome and intestinal permeability (leaky gut syndrome) could play a role in the development or exacerbation of the condition. Glyphosate, as an endocrine disruptor and a substance potentially harmful to gut health, has been suggested as a possible environmental factor that could contribute to PCOS development or severity [77].

The role of genetics in polycystic ovary syndrome (PCOS) remains complex and not fully understood. While researchers have identified certain genetic variants associated with PCOS, these genetic factors alone do not account for the majority of cases. Indeed, many women with PCOS do not exhibit clear genetic mutations that can explain the development of the condition. Environmental factors, including exposure to certain chemicals and toxins, have been proposed as potential contributors to the development of PCOS. Glyphosate, the active ingredient in Roundup and other herbicides, has been implicated as one such environmental factor. Studies have shown that glyphosate can disrupt the activity of enzymes involved in steroid metabolism, including hexose-6-phosphate dehydrogenase (H6PD), aromatase, and PAPS synthase. These enzymes play important roles in hormone regulation and metabolism, and their dysregulation has been associated with PCOS. Glyphosate’s ability to substitute for glycine in these enzymes may impair their function, potentially leading to the hormonal imbalances characteristic of PCOS. Overall, while genetics may play a role in predisposing individuals to PCOS, environmental factors such as glyphosate exposure may also contribute to the development and manifestation of the condition. Further research is needed to fully elucidate the interplay between genetic susceptibility and environmental influences in PCOS pathogenesis [74,78].

## 5. Challenges of Glyphosate Use in Achieving Sustainable Development Goals—SDGs

Sustainability is a complex concept often associated with humans coexisting harmoniously with nature or a system that supports people, planet, and profit. The most comprehensive definition of sustainability encompasses practices that enable the current population to fulfill its basic needs without compromising the needs of future generations [79]. In 2015, the United Nations adopted the Sustainable Development Goals (SDGs), also known as the Global Goals, to universally encourage efforts to eradicate poverty, safeguard the environment, and promote global peace and prosperity for all by 2030. Seventeen SDGs were integrated, recognizing that action in one area can impact outcomes in others, and that development must strike a balance between social, economic, and environmental sustainability [80].

**Figure 2:**
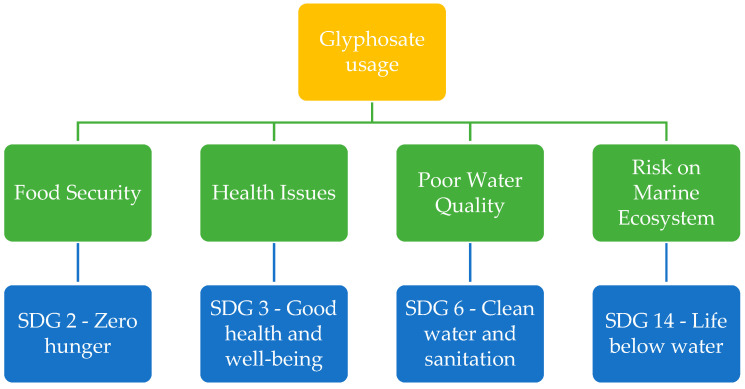
Glyphosate usage and its direct and indirect impact on the Sustainable Development Goals.

The use of glyphosate can be linked to various Sustainable Development Goals (SDGs), particularly those related to environmental sustainability, health, and agriculture (Figure 2). This connection has the potential to hinder progress towards these goals and delay their achievement.

### 5.1. SDG 2—Zero Hunger

SDG 2 focuses on ending hunger, achieving food security, improving nutrition, and promoting sustainable agriculture to ensure that everyone has access to sufficient and nutritious food [81]. While glyphosate can contribute to efficient food production, concerns exist about its impact on soil health, biodiversity, and potential residues in food [4]. The sustainable use of glyphosate and other agricultural practices that promote soil health and ecosystem resilience are crucial for achieving zero hunger (SDG 2).

### 5.2. SDG 3—Good Health and Well-Being

SDG 3 aims to ensure healthy lives and promote well-being for all at all ages by improving healthcare services and addressing major health issues [82]. As highlighted previously, glyphosate exposure has been linked to several health issues, including but not limited to gut dysbiosis, autism, liver dysfunctions, respiratory issues, and reproductive problems. Achieving good health and well-being (SDG 3) requires creating safe and healthy environments that support well-being, and the use of glyphosate may pose risks to this objective.

### 5.3. SDG 6—Clean Water and Sanitation

SDG 6 emphasizes the importance of ensuring clean and accessible water for all, thereby improving hygiene, health, and overall living conditions [83]. Glyphosate can enter water bodies through runoff or leaching, potentially contaminating water sources [23]. This contamination poses risks to water quality. Addressing glyphosate use and its impact on water resources is important for achieving clean water and sanitation (SDG 6).

### 5.4. SDG 14—Life Below Water

SDG 14 aims to conserve and sustainably use the oceans, seas, and marine resources for sustainable development [84]. The use of glyphosate in agriculture poses a significant risk to marine ecosystems. When applied, glyphosate contaminates not only the soil but also water bodies through runoff and leaching, which can have detrimental effects on aquatic life [23].

## 6. Glyphosate and Organic Food

Legally, glyphosate is prohibited for use on certified organic crops. However, organic certification does not guarantee that a product is entirely free from glyphosate. While organic foods typically show considerably lower levels of glyphosate compared to conventionally grown counterparts, avoiding glyphosate entirely is exceedingly challenging due to its presence in soil, animal manure, rainwater, and airborne drift. The widespread use of glyphosate-based herbicides allows for contamination, even in food produced far from treated areas. Individuals who primarily consume organic foods exhibit notably lower levels of glyphosate in their urine compared to those who predominantly consume conventional foods. Moreover, individuals in good health tend to exhibit significantly lower glyphosate levels in their urine compared to those with chronic illnesses. Nevertheless, achieving complete avoidance of glyphosate remains highly challenging [1].

## 7. Glycine a Potential Neutralization Substance for Glyphosate in the Body

Glyphosate has the potential to replace glycine in various crucial proteins, leading to their dysfunction. This phenomenon contributes significantly to the link between glyphosate exposure and the rise in various diseases. Glycine, the simplest amino acid with just a single hydrogen atom as its side chain, plays vital roles in proteins requiring flexibility or the ability to bind to bulky substrates. Each amino acid, including glycine, is encoded by specific three-letter sequences in the DNA code. When glycine is needed, its specific transfer RNA (tRNA) synthetase matches the code to the corresponding glycine molecule, ensuring precise protein synthesis [1].

**Figure 3:**
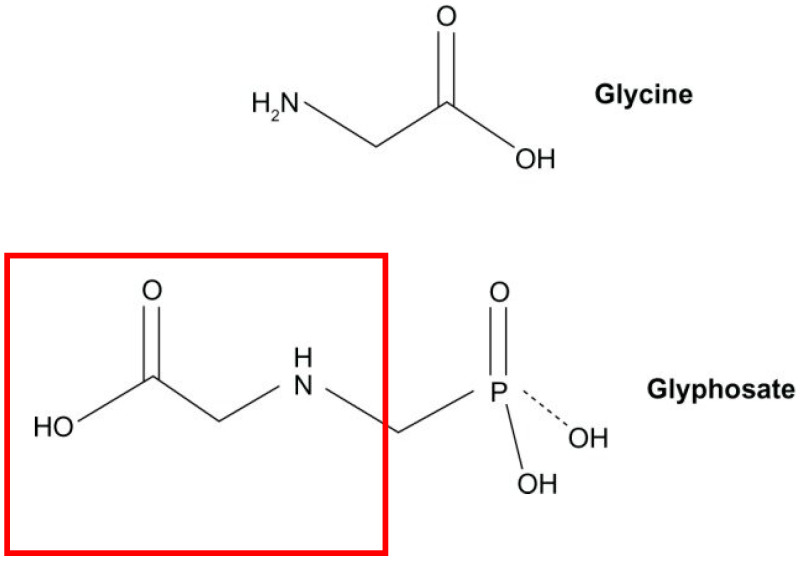
Similarities between the chemical structures of Glycine and Glyphosate.

Glyphosate, sharing similarities with glycine (Figure 3), can fit into the pocket meant for glycine in proteins. However, its additional molecular group attached to its nitrogen atom, while not technically a side chain, may hinder its substitution in certain cases, depending on neighboring amino acids. Nonetheless, glyphosate’s ability to fit into the glycine-binding pocket allows it to interfere with normal protein function. This is exemplified in plants, where glyphosate disrupts the action of the enzyme EPSP synthase, typically containing a glycine residue preceded by an alanine at the site of disruption [1]. The consequences of glyphosate’s ability to integrate into proteins in place of the coding amino acid glycine are extensive and potentially catastrophic. Nearly all proteins contain at least one glycine residue, and there is a substantial subset of proteins where a specific glycine residue is crucial for proper functionality. This disruption in protein structure and function underscores the far-reaching impacts of glyphosate exposure on biological systems [1].

One potential approach to counteract glyphosate’s impact on the body involves the amino acid glycine. Glycine, abundant in protein-rich foods, may serve as a competitor to glyphosate within the body. By consuming adequate amounts of glycine, individuals may enhance their body’s ability to neutralize excess glyphosate, potentially mitigating its adverse effects. This warrants further exploration into dietary interventions to counteract glyphosate exposure.

## 8. Alternatives to Glyphosate

Weed interference significantly reduces crop yields, impacting global food production. Weeds compete with crops for resources such as water, light, and nutrients, and can also produce substances that inhibit crop growth. Managing weeds has been a longstanding challenge for farmers since the beginning of agriculture. In earlier times, before synthetic chemicals were available, weed control relied on methods such as manual weeding, crop rotation, polyculture, and other sustainable management practices with low input requirements [85]. Physical weed control methods, including soil heating, mulching, and covering, are also effective strategies [86].

**Figure 4:**
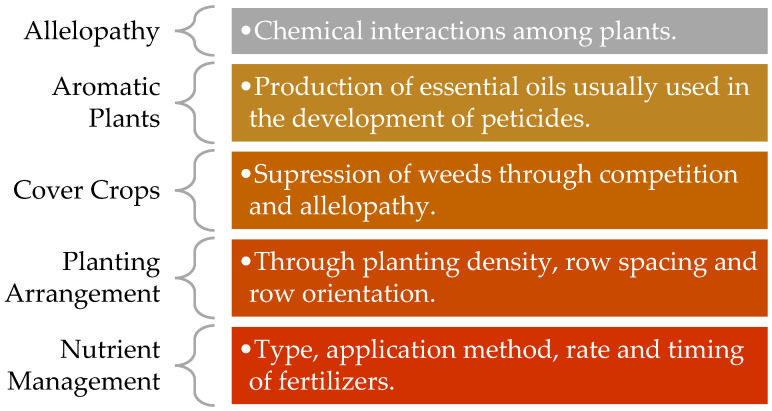
Alternative approaches for weed control.

Cover crops are plants grown primarily to protect and improve soil. They reduce erosion, suppress weeds, enhance soil structure, and can influence nutrient cycling, in addition to their roles in glyphosate management (Figure 4).

Building on these physical and sustainable practices, we can identify seven categories of non-chemical, organic, and natural herbicides. These alternatives have lower toxicity compared to glyphosate, although they are often significantly more expensive and may exhibit variations in quality between batches. The seven categories include:

(1) Natural acids—combination of vinegar and citric acids.
(2) Herbicidal soaps.
(3) Salt-based herbicides.
(4) Iron-based herbicides.
(5) Phytotoxic oils such as peppermint, pine, clove, and citronella.
(6) Corn gluten.
(7) Combination products including ingredients from the six other categories.

Although these alternative products are not as efficient as glyphosate, their combined use can have synergistic effects when integrated with good agricultural practices, such as improving soil health, irrigation, cultivar selection, proper moving, avoiding conditions that favor weed growth, such as non-optimal soil pH, overwatering, excessive nitrogen application and other factors [87]. However, large-scale agricultural production will likely continue to rely on these chemical aids to maintain crop yields, particularly as the world population grows and weed resistance to individual herbicides remains limited.

## 9. Conclusions

In conclusion, glyphosate is pervasive in our food, air and water, affecting both human and environmental health. Its association with various diseases, including reproductive, metabolic, neurological, oncological, and autoimmune disorders such as autism, depression, polycystic ovary syndrome, infertility, thyroid dysfunction, respiratory infections, and gut dysbiosis, underscores the urgent need for action. In addition, glyphosate has the potential to hinder the achievement of certain Sustainable Development Goals, including Zero Hunger (SDG 2), Good Health and Well-being (SDG 3), Clean Water and Sanitation (SDG 6), and Life Below Water (SDG 14). To safeguard public health and environmental sustainability, it is imperative to adopt safer and more sustainable agricultural practices that improve soil and crop health without negatively impacting our environment, infrastructure, and overall well-being. Importantly, several areas remain inconclusive and require further investigation, including the long-term effects on soil microbiota, potential subtle human health impacts at environmental exposure levels, ecological effects on non-target organisms such as earthworms, and the efficiency and safety of alternative herbicide strategies. By summarizing these knowledge gaps, we aim to provide a balanced overview and guide future research priorities.

## Data Availability

No new data were created or analyzed in this study. Data sharing is not applicable to this article.

## References

[1] Seneff S. (2021). Toxic Legacy: How the Weedkiller Glyphosate Is Destroying Our Health and the Environment.

[2] Singh S., Kumar V., Datta S., Wani A.B., Dhanjal D.S., Romero R., Singh J. (2020). Glyphosate Uptake, Translocation, Resistance Emergence in Crops, Analytical Monitoring, Toxicity and Degradation: A Review. Environ. Chem. Lett..

[3] Baylis A. (2020). Why Glyphosate Is a Global Herbicide: Strengths, Weaknesses and Prospects. Pest Manag. Sci..

[4] Marques J.G.D.C., Veríssimo K.J.D.S., Fernandes B.S., Ferreira S.R.D.M., Montenegro S.M.G.L., Motteran F. (2021). Glyphosate: A Review on the Current Environmental Impacts from a Brazilian Perspective. Bull. Environ. Contam. Toxicol..

[5] Soares D., Silva L., Duarte S., Pena A., Pereira A. (2021). Glyphosate Use, Toxicity and Occurrence in Food. Foods.

[6] De Castilhos Ghisi N., Zuanazzi N.R., Fabrin T.M.C., Oliveira E.C. (2020). Glyphosate and Its Toxicology: A Scientometric Review. Sci. Total Environ..

[7] Lacroix R., Kurrasch D.M. (2023). Glyphosate Toxicity: In Vivo, in Vitro, and Epidemiological Evidence. Toxicol. Sci..

[8] McHenry L.B. (2018). The Monsanto Papers: Poisoning the Scientific Well. Int. J. Risk Saf. Med..

[9] Williams G.M., Aardema M., Acquavella J., Berry S.C., Brusick D., Burns M.M., De Camargo J.L.V., Garabrant D., Greim H.A., Kier L.D. (2016). A Review of the Carcinogenic Potential of Glyphosate by Four Independent Expert Panels and Comparison to the IARC Assessment. Crit. Rev. Toxicol..

[10] Sharma S.D., Singh M. (2001). Environmental Factors Affecting Absorption and Bio-Efficacy of Glyphosate in Florida Beggarweed (Desmodium Tortuosum). Crop Prot..

[11] Rueppel M.L., Brightwell B.B., Schaefer J., Marvel J.T. (1977). Metabolism and Degradation of Glyphosate in Soil and Water. J. Agric. Food Chem..

[12] Lancaster S.H., Hollister E.B., Senseman S.A., Gentry T.J. (2010). Effects of Repeated Glyphosate Applications on Soil Microbial Community Composition and the Mineralization of Glyphosate: Soil Microbial Response to Repeated Glyphosate Applications. Pest Manag. Sci..

[13] Stenrod M., Eklo O.M., Charnay M., Benoit P. (2005). Effect of Freezing and Thawing on Microbial Activity and Glyphosate Degradation in Two Norwegian Soils. Pest Manag. Sci..

[14] Zaranyika M.F., Nyandoro M.G. (1993). Degradation of Glyphosate in the Aquatic Environment: An Enzymic Kinetic Model That Takes into Account Microbial Degradation of Both Free and Colloidal (or Sediment) Particle Adsorbed Glyphosate. J. Agric. Food Chem..

[15] Sorensen S.R., Schultz A., Jacobsen O.S., Aamand J. (2006). Sorption, Desorption and Mineralisation of the Herbicides Glyphosate and MCPA in Samples from Two Danish Soil and Subsurface Profiles. Environ. Pollut..

[16] Schnürer Y., Persson P., Nilsson M., Nordgren A., Giesler R. (2006). Effects of Surface Sorption on Microbial Degradation of Glyphosate. Environ. Sci. Technol..

[17] Glass R.L. (1987). Adsorption of Glyphosate by Soils and Clay Minerals. J. Agric. Food Chem..

[18] Mesnage R., Defarge N., Spiroux De Vendômois J., Séralini G.E. (2015). Potential Toxic Effects of Glyphosate and Its Commercial Formulations below Regulatory Limits. Food Chem. Toxicol..

[19] Peillex C., Pelletier M. (2020). The Impact and Toxicity of Glyphosate and Glyphosate-Based Herbicides on Health and Immunity. J. Immunotoxicol..

[20] Gravena R., Filho R.V., Alves P.L.C.A., Mazzafera P., Gravena A.R. (2012). Glyphosate Has Low Toxicity to Citrus Plants Growing in the Field. Can. J. Plant Sci..

[21] Liu Y., Tikunov Y., Schouten R.E., Marcelis L.F.M., Visser R.G.F., Bovy A. (2018). Anthocyanin Biosynthesis and Degradation Mechanisms in Solanaceous Vegetables: A Review. Front. Chem..

[22] Funke T., Han H., Healy-Fried M.L., Fischer M., Schönbrunn E. (2006). Molecular Basis for the Herbicide Resistance of Roundup Ready Crops. Proc. Natl. Acad. Sci. USA.

[23] Kanissery R., Gairhe B., Kadyampakeni D., Batuman O., Alferez F. (2019). Glyphosate: Its Environmental Persistence and Impact on Crop Health and Nutrition. Plants.

[24] Chang S.Y., Liao C.-H. (2002). Analysis of Glyphosate, Glufosinate and Aminomethylphosphonic Acid by Capillary Electrophoresis with Indirect Fluorescence Detection. J. Chromatogr. A.

[25] Al-Khatib K., Peterson D. (1999). Soybean (Glycine Max) Response to Simulated Drift from Selected Sulfonylurea Herbicides, Dicamba, Glyphosate, and Glufosinate. Weed Technol..

[26] Ellis J.M., Griffin J.L. (2002). Soybean (Glycine Max) and Cotton (Gossypium Hirsutum) Response to Simulated Drift of Glyphosate and Glufosinate. Weed Technol..

[27] Reddy K.N., Rimando A.M., Duke S.O. (2004). Aminomethylphosphonic Acid, a Metabolite of Glyphosate, Causes Injury in Glyphosate-Treated, Glyphosate-Resistant Soybean. J. Agric. Food Chem..

[28] Frank J.E., Mao M.K., Sikorski J.A. (1997). Glyphosate: A Unique Global Herbicide.

[29] Johal G.S., Rahe J.E. (1988). Glyphosate, Hypersensitivity and Phytoalexin Accumulation in the Incompatible Bean Anthracnose Host-Parasite Interaction. Physiol. Mol. Plant Pathol..

[30] Yamada T., Kremer R.J., De Camargo E Castro P.R., Wood B.W. (2009). Glyphosate Interactions with Physiology, Nutrition, and Diseases of Plants: Threat to Agricultural Sustainability?. Eur. J. Agron..

[31] Lévesque C.A., Rahe J.E., Eaves D.M. (1987). Effects of Glyphosate on *Fusarium* spp.: Its Influence on Root Colonization of Weeds, Propagule Density in the Soil, and Crop Emergence. Can. J. Microbiol..

[32] Duke S.O., Powles S.B. (2008). Glyphosate: A Once-in-a-century Herbicide. Pest Manag. Sci..

[33] Kanissery R.G., Welsh A., Sims G.K. (2015). Effect of Soil Aeration and Phosphate Addition on the Microbial Bioavailability of Carbon-14-Glyphosate. J. Environ. Qual..

[34] Duke S.O., Lydon J., Koskinen W.C., Moorman T.B., Chaney R.L., Hammerschmidt R. (2012). Glyphosate Effects on Plant Mineral Nutrition, Crop Rhizosphere Microbiota, and Plant Disease in Glyphosate-Resistant Crops. J. Agric. Food Chem..

[35] Wood L.J., Botten N., Fredeen A.L., Werner J.R. (2024). Glyphosate-Based Herbicide Contributes to Nutrient Variability in Forest Plants. Front. For. Glob. Change.

[36] Klátyik S., Simon G., Takács E., Oláh M., Zaller J.G., Antoniou M.N., Benbrook C., Mesnage R., Székács A. (2025). Toxicological Concerns Regarding Glyphosate, Its Formulations, and Co-Formulants as Environmental Pollutants: A Review of Published Studies from 2010 to 2025. Arch. Toxicol..

[37] Cakmak I., Yazici A., Tutus Y., Ozturk L. (2009). Glyphosate Reduced Seed and Leaf Concentrations of Calcium, Manganese, Magnesium, and Iron in Non-Glyphosate Resistant Soybean. Eur. J. Agron..

[38] Gimsing A.L., Borggaard O.K. (2002). Competitive Adsorption and Desorption of Glyphosate and Phosphate on Clay Silicates and Oxides. Clay Miner..

[39] Schütte G., Eckerstorfer M., Rastelli V., Reichenbecher W., Restrepo-Vassalli S., Ruohonen-Lehto M., Saucy A.-G.W., Mertens M. (2017). Herbicide Resistance and Biodiversity: Agronomic and Environmental Aspects of Genetically Modified Herbicide-Resistant Plants. Environ. Sci. Eur..

[40] Mamy L., Barriuso E., Gabrielle B. (2016). Glyphosate Fate in Soils When Arriving in Plant Residues. Chemosphere.

[41] Vereecken H. (2005). Mobility and Leaching of Glyphosate: A Review. Pest Manag. Sci..

[42] Benbrook C.M. (2016). Trends in Glyphosate Herbicide Use in the United States and Globally. Environ. Sci. Eur..

[43] Agostini L.P., Dettogni R.S., Dos Reis R.S., Stur E., Dos Santos E.V.W., Ventorim D.P., Garcia F.M., Cardoso R.C., Graceli J.B., Louro I.D. (2020). Effects of Glyphosate Exposure on Human Health: Insights from Epidemiological and in Vitro Studies. Sci. Total Environ..

[44] Gaupp-Berghausen M., Hofer M., Rewald B., Zaller J.G. (2015). Glyphosate-Based Herbicides Reduce the Activity and Reproduction of Earthworms and Lead to Increased Soil Nutrient Concentrations. Sci. Rep..

[45] Bai S.H., Ogbourne S.M. (2016). Glyphosate: Environmental Contamination, Toxicity and Potential Risks to Human Health via Food Contamination. Environ. Sci. Pollut. Res..

[46] Fréville M., Henri J., Estienne A., Serra L., Ramé C., Ganier P., Chahnamian M., Froment P., Dupont J. (2023). Determination of the Elimination Half-Life of Glyphosate and Its Main Metabolite, AMPA, in Chicken Plasma. Toxicol. Lett..

[47] Duke S.O. (2020). Glyphosate: Environmental Fate and Impact. Weed Sci..

[48] Newmark H.L., Lupton J.R. (1990). Determinants and Consequences of Colonic Luminal pH: Implications for Colon Cancer. Nutr. Cancer.

[49] Nakae H., Kusanagi M., Okuyama M., Igarashi T. (2015). Paralytic Ileus Induced by Glyphosate Intoxication Successfully Treated Using Kampo Medicine. Acute Med. Surg..

[50] Mao Q., Manservisi F., Panzacchi S., Mandrioli D., Menghetti I., Vornoli A., Bua L., Falcioni L., Lesseur C., Chen J. (2018). The Ramazzini Institute 13-Week Pilot Study on Glyphosate and Roundup Administered at Human-Equivalent Dose to Sprague Dawley Rats: Effects on the Microbiome. Environ. Health.

[51] Fausto N., Campbell J.S., Riehle K.J. (2012). Liver Regeneration. J. Hepatol..

[52] Fathi M.A., Han G., Kang R., Shen D., Shen J., Li C. (2020). Disruption of Cytochrome P450 Enzymes in the Liver and Small Intestine in Chicken Embryos in Ovo Exposed to Glyphosate. Environ. Sci. Pollut. Res..

[53] Mills P.J., Caussy C., Loomba R. (2020). Glyphosate Excretion Is Associated With Steatohepatitis and Advanced Liver Fibrosis in Patients With Fatty Liver Disease. Clin. Gastroenterol. Hepatol..

[54] Martínez M.-A., Ares I., Rodríguez J.-L., Martínez M., Martínez-Larrañaga M.-R., Anadón A. (2018). Neurotransmitter Changes in Rat Brain Regions Following Glyphosate Exposure. Environ. Res..

[55] Cattani D., De Liz Oliveira Cavalli V.L., Heinz Rieg C.E., Domingues J.T., Dal-Cim T., Tasca C.I., Mena Barreto Silva F.R., Zamoner A. (2014). Mechanisms Underlying the Neurotoxicity Induced by Glyphosate-Based Herbicide in Immature Rat Hippocampus: Involvement of Glutamate Excitotoxicity. Toxicology.

[56] Masood M.I., Naseem M., Warda S.A., Tapia-Laliena M.Á., Rehman H.U., Nasim M.J., Schäfer K.H. (2021). Environment Permissible Concentrations of Glyphosate in Drinking Water Can Influence the Fate of Neural Stem Cells from the Subventricular Zone of the Postnatal Mouse. Environ. Pollut..

[57] Rueda-Ruzafa L., Cruz F., Roman P., Cardona D. (2019). Gut Microbiota and Neurological Effects of Glyphosate. NeuroToxicology.

[58] Chaste P., Leboyer M. (2012). Autism Risk Factors: Genes, Environment, and Gene-Environment Interactions. Dialogues Clin. Neurosci..

[59] Gherardi R.K., Eidi H., Crapeaux G., Authier F.J., Cadusseau J. (2015). Biopersistence and Brain Translocation of Aluminum Adjuvants of Vaccines. Front. Neurol..

[60] Seneff S., Swanson N., Li C. (2015). Aluminum and Glyphosate Can Synergistically Induce Pineal Gland Pathology: Connection to Gut Dysbiosis and Neurological Disease. Agric. Sci..

[61] Von Ehrenstein O.S., Ling C., Cui X., Cockburn M., Park A.S., Yu F., Wu J., Ritz B. (2019). Prenatal and Infant Exposure to Ambient Pesticides and Autism Spectrum Disorder in Children: Population Based Case-Control Study. BMJ.

[62] Dallegrave E., Mantese F.D., Coelho R.S., Pereira J.D., Dalsenter P.R., Langeloh A. (2003). The Teratogenic Potential of the Herbicide Glyphosate-Roundup^®^ in Wistar Rats. Toxicol. Lett..

[63] Rattan S., Zhou C., Chiang C., Mahalingam S., Brehm E., Flaws J.A. (2017). Exposure to Endocrine Disruptors during Adulthood: Consequences for Female Fertility. J. Endocrinol..

[64] Vandenberg L.N., Colborn T., Hayes T.B., Heindel J.J., Jacobs D.R., Lee D.-H., Shioda T., Soto A.M., Vom Saal F.S., Welshons W.V. (2012). Hormones and Endocrine-Disrupting Chemicals: Low-Dose Effects and Nonmonotonic Dose Responses. Endocr. Rev..

[65] Anifandis G., Amiridis G., Dafopoulos K., Daponte A., Dovolou E., Gavriil E., Gorgogietas V., Kachpani E., Mamuris Z., Messini C. (2017). The In Vitro Impact of the Herbicide Roundup on Human Sperm Motility and Sperm Mitochondria. Toxics.

[66] Canosa I.S., Zanitti M., Lonné N., Medesani D.A., López Greco L.S., Rodríguez E.M. (2019). Imbalances in the Male Reproductive Function of the Estuarine Crab Neohelice Granulata, Caused by Glyphosate. Ecotoxicol. Environ. Saf..

[67] Walsh L.P., McCormick C., Martin C., Stocco D.M. (2000). Roundup Inhibits Steroidogenesis by Disrupting Steroidogenic Acute Regulatory (StAR) Protein Expression. Environ. Health Perspect..

[68] Richard S., Moslemi S., Sipahutar H., Benachour N., Seralini G.-E. (2005). Differential Effects of Glyphosate and Roundup on Human Placental Cells and Aromatase. Environ. Health Perspect..

[69] Parvez S., Gerona R.R., Proctor C., Friesen M., Ashby J.L., Reiter J.L., Lui Z., Winchester P.D. (2018). Glyphosate Exposure in Pregnancy and Shortened Gestational Length: A Prospective Indiana Birth Cohort Study. Environ. Health.

[70] De Oliveira M.A.L., Rojas V.C.T., De Sá J.C., De Novais C.O., Silva M.S., De Almeida Paula H.A., Kirsten T.B., Bernardi M.M., Pinheiro L.C., Giusti-Paiva A. (2022). Perinatal Exposure to Glyphosate-based Herbicides Induced Neurodevelopmental Behaviors Impairments and Increased Oxidative Stress in the Prefrontal Cortex and Hippocampus in Offspring. Int. J. Dev. Neurosci..

[71] Kongtip P., Nankongnab N., Phupancharoensuk R., Palarach C., Sujirarat D., Sangprasert S., Sermsuk M., Sawattrakool N., Woskie S.R. (2017). Glyphosate and Paraquat in Maternal and Fetal Serums in Thai Women. J. Agromedicine.

[72] Shrestha S., Parks C.G., Goldner W.S., Kamel F., Umbach D.M., Ward M.H., Lerro C.C., Koutros S., Hofmann J.N., Beane Freeman L.E. (2018). Pesticide Use and Incident Hypothyroidism in Pesticide Applicators in the Agricultural Health Study. Environ. Health Perspect..

[73] Sirmans S., Pate K. (2013). Epidemiology, Diagnosis, and Management of Polycystic Ovary Syndrome. Clin. Epidemiol..

[74] Chen J., Shen S., Tan Y., Xia D., Xia Y., Cao Y., Wang W., Wu X., Wang H., Yi L. (2015). The Correlation of Aromatase Activity and Obesity in Women with or without Polycystic Ovary Syndrome. J. Ovarian Res..

[75] Hakim C., Padmanabhan V., Vyas A.K. (2017). Gestational Hyperandrogenism in Developmental Programming. Endocrinology.

[76] Cherskov A., Pohl A., Allison C., Zhang H., Payne R.A., Baron-Cohen S. (2018). Polycystic Ovary Syndrome and Autism: A Test of the Prenatal Sex Steroid Theory. Transl. Psychiatry.

[77] Parker J. (2015). A New Hypothesis for the Mechanism of Glyphosate Induced Intestinal Permeability in the Pathogenesis of Polycystic Ovary Syndrome. J. Australas. Coll. Nutr. Environ. Med..

[78] Oostdijk W., Idkowiak J., Mueller J.W., House P.J., Taylor A.E., O’Reilly M.W., Hughes B.A., De Vries M.C., Kant S.G., Santen G.W.E. (2015). PAPSS2 Deficiency Causes Androgen Excess via Impaired DHEA Sulfation—In Vitro and in Vivo Studies in a Family Harboring Two Novel PAPSS2 Mutations. J. Clin. Endocrinol. Metab..

[79] Henderson K., Loreau M. (2023). A Model of Sustainable Development Goals: Challenges and Opportunities in Promoting Human Well-Being and Environmental Sustainability. Ecol. Model..

[80] United Nations Development Programme (2023). The Sustainable Development Goals Report.

[81] Schwindenhammer S., Partzsch L., Partzsch L. (2023). SDG 2 and the Dominance of Food Security in the Global Agri-Food Norm Cluster. The Environment in Global Sustainability Governance.

[82] Martelletti P., Leonardi M., Ashina M., Burstein R., Cho S.-J., Charway-Felli A., Dodick D.W., Gil-Gouveia R., Grazzi L., Lampl C. (2023). Rethinking Headache as a Global Public Health Case Model for Reaching the SDG 3 HEALTH by 2030. J. Headache Pain.

[83] Hellberg S., Söderbaum F., Swain A., Öjendal J. (2023). Strategies Towards SDG 6 Implementation. Routledge Handbook of Water and Development.

[84] Vadrot A.B.M., Partzsch L. (2023). Protecting Life below Water: Competing Normative, Economic and Epistemic Orders (SDG 14). The Environment in Global Sustainability Governance.

[85] Mohammadi G.R., Soloneski S. (2013). Alternative Weed Control Methods: A Review. Weed and Pest Control—Conventional and New Challenges.

[86] Gao W.-T., Su W.-H. (2024). Weed Management Methods for Herbaceous Field Crops: A Review. Agronomy.

[87] Deborah S.-F., Stanton G. (2024). Vinegar: An Alternative to Glyphosate?.

